# Supplementary material for: A novel interaction between the 5′ untranslated region of the Chikungunya virus genome and Musashi RNA binding protein is essential for efficient virus genome replication
Source: Nucleic Acids Res. 2024 Aug 1;52(17):10654–67. doi: 10.1093/nar/gkae619 (PMC11417370; doi:10.1093/nar/gkae619)

Supplementary data 1: Alignment of CHIKV 5’ region (nts 61-120) showing variation within 5’UTR MSI-2 binding site. All complete CHIKV genomes were downloaded from the NCBI, by using the selection for complete genomes and search term for CHIKV. Duplicates were removed, leaving one representative of each, which were aligned using CLUSTAL OMEGA (multiple sequence alignment) on EBI default settings. MSI-2 binding site indicated by orange box.

```
Accession number                    61                                                        120
gi|1032899617|gb|KX262992.1|        AGATTAATAACCCATCATGGATTCTGTGTACGTGGATATAGACGCTGACAGCGCCTTTTT
gi|1168909638|gb|KY703980.1|        AGATTAATAACCCATCATGGATTCTGTGTACGTGGATATAGACGCTGACAGCGCCTTTTT
gi|2316015176|gb|MZ703085.1|        AGATTAAGAACCCATCATGGATCCTGTGTACGTGGACATAGACGCTGACAGCGCCTTTTT
gi|1032899611|gb|KX262991.1|        AGATTAATAACCCATCATGGATTCTGTGTACGTGGATATAGACGCTGACAGCGCCTTTTT
gi|674268615|gb|KF590565.1|         AGATTAATAACCCATCATGGATCCTGTGTACGTGGACATAGACGCTGACAGCGCCTTTTT
gi|2322557117|gb|OK562602.1|        AGATTAAGAACCCATCATGGATCCTGTGTACGTGGACATAGACGCTGACAGCGCCTTTTT
gi|1781339200|gb|MK163628.1|        AGATTAATAACCCATCATGGATCCTGTGTACGTGGACATAGACGCTGACAGCGCCTTTTT
gi|262073953|gb|FJ513632.1|         AGATTAATAACCCATCATGGATCCTGTGTACGTGGACATAGACGCCGACAGCGCCTTTTT
gi|674268618|gb|KF590566.1|         AGATTAATAACCCATCATGGATCCTGTGTACGTGGACATAGACGCTGACAGCGCCTTTTT
gi|1050242909|gb|KT324228.1|        AGATTAATAACCCATCATGGATCCTGTGTACGTGGACATAGACGCTGACAGCGCCTTTTT
gi|655168280|gb|KJ796847.1|         AGATTAATAACCCATCATGGATCCTGTGTACGTGGACATAGACGCTGACAGCGCCTTTTT
gi|629510202|dbj|AB860301.3|        AGATTAATAACCCATCATGGATTCTGTGTACGTGGATATAGACGCTGACAGCGCCTTTTT
gi|429324006|emb|FN295483.3|        AGATCAATAACCCATCATGGATTCTGTGTACGTGGATATAGACGCTGACAGCGCCTTTTT
gi|1655475674|gb|MK690206.1|        AGATTAAGAACCCATCATGGATCCTGTGTACGTGGACATAGACGCTGACAGCGCCTTTTT
gi|410067116|emb|FR717336.1|        AGATTAATAACCCATCATGGATCCTGTGTACGTGGACATAGACGCTGACAGCGCCTTTTT
gi|2484235404|gb|OQ605447.1|        AGATTAATAACCCATCATGGATCCTGTGCACGTGGACATAGACGCTGACAGCGCCTTTTT
gi|1168909306|gb|KY703922.1|        AGATTAATAACCCATCATGGATTCTGTGTACGTGGATATAGACGCTGACAGCGCCTTTTT
gi|655168286|gb|KJ796850.1|         AGATTAATAACCCATCATGGATCCTGTGTACGTGGACATAGACGCTGACAGCGCCTTTTT
gi|1240405768|gb|MF499120.1|        AGATTAATAACCCATCATGGATCCTGTGTACGTGGACATAGACGCTGACAGCGCCTTTTT
gi|2186567288|gb|OL979153.1|        AGATTAATAACCCATCATGGATCCTGTGTACGTGGATATAGACGCTGACAGCGCCTTTTT
gi|27734686|gb|AF369024.2|          AGATTAAGAACCCATCATGGATCCTGTGTACGTGGACATAGACGCTGACAGCGCCTTTTT
gi|189409703|gb|EU703760.1|         AGATCAATAACCCATCATGGATTCTGTGTACGTGGATATAGACGCTGACAGCGCCTTTTT
gi|1189411909|gb|MF001514.1|        AGATTAATAACCCATCATGGATTCTGTGTACGTGGATATAGACGCTGACAGCGCCTTTTT
gi|2186567368|gb|OL999095.1|        AGATTAATAACCCATCATGGATCCTGTGTACGTGGACATAGACGCTGACAGCGCCTTTTT
gi|1391207960|gb|MG921596.1|        AGATTAATAACCCATCATGGATTCTGTGTACGTGGATATAGACGCTGACAGCGCCTTTTT
gi|615794500|gb|KJ451622.1|         AGATTAATAACCCATCATGGATTCTGTGTACGTGGATATAGACGCTGACAGCGCCTTTTT
gi|1189411921|gb|MF001518.1|        AGATTAATAACCCATCATGGATTCTGTGTACGTGGATATAGACGCTGACAGCGCCTTTTT
gi|1796551930|gb|MN402890.1|        AGATTAATAACCCATCATGGATCCTGTGTACGTGGATATAGACGCTGACAGCGCCTTTTT
gi|288572686|gb|FJ445427.2|         AGATTAATAACCCATCATGGATCCTGTGTACGTGGACATAGACGCTGACAGCGCCTTCTT
gi|1967465569|gb|MW042254.1|        AGATTAATAACCCATCATGGATCCTGTGTACGTGGACATAGACACTGACAGCGCCTTTTT
gi|1621066037|gb|MK473621.1|        AGATTAATAACCCATCATGGATCCTGTGTACGTGGACATAGACGCTGACAGCGCCTTTTT
gi|262410949|gb|FJ807897.1|         AGATTAATAACCCATCATGGATTCTGTGTACGTGGATATAGACGCTGACAGCGCCTTTTT
gi|2484235419|gb|OQ605452.1|        AGATTAACAACCCATCATGGATCCTGTGCACGTGGACATAGACGCTGACAGCGCCTTTTT
gi|2484235374|gb|OQ605437.1|        AGATTAACAACCCATCATGGATCCTGTGTACGTGGACATAGACGCTGACAGCGCCTTTTT
gi|262073950|gb|FJ513629.1|         AGATTAATAACCCATCATGGATCCTGTGTACGTGGACATAGACGCTGACAGCGCCTTTTT
gi|1677557230|gb|MH329296.1|        AGATTAATAACCCATCATGGATTCTGTGTACGTGGATATAGACGCTGACAGCGCCTTTTT
gi|745698469|gb|KP003807.1|         AGATTAATAACCCATCATGGATCCTGTGTACGTGGACATAGACGCTGACAGCGCCTTTTT
gi|564970356|gb|KC614648.1|         AGATTAATAACCCATCATGGATCCTGTGTACGTGGACATAGACGCTGACAGCGCCTTTTT
gi|2563904640|gb|OR037307.1|        AGATTAATAACCCATCATGGATCCTGTGTACGTGGATGTAGACGCTGACAGCGCCTTTTT
gi|189409706|gb|EU703761.1|         AGATCAATAACCCATCATGGATTCTGTGTACGTGGATATAGACGCTGACAGCGCCTTTTT
gi|133779733|gb|EF452494.1|         AGATTAATAACCCATCATGGATTCTGTGTACGTGGACATAGACGCTGACAGCGCCTTTTT
gi|1021315749|gb|KT308159.1|        AGATTAATAACCCATCATGGATTCTGTGTACGTGGATATAGACGCTGACAGCGCCTTTTT
gi|1168909620|gb|KY703977.1|        AGATTAATAACCCATCATGGATTCTGTGTACGTGGATATAGACGCTGACAGCGCCTTTTT
gi|1189411906|gb|MF001513.1|        AGATTAATAACCCATCATGGATTCTGTGTACGTGGATATAGACGCTGACAGCGCCTTTTT
gi|1189411897|gb|MF001510.1|        AGATTAATAACCCATCATGGATTCTGTGTACGTGGATATAGACGCTGACAGCGCCTTTTT
gi|1189411903|gb|MF001512.1|        AGATTAATAACCCATCATGGATTCTGTGTACGTGGATATAGACGCTGACAGCGCCTTTTT
gi|1168909711|gb|KY703992.1|        AGATTAATAACCCATCATGGATTCTGTGTACGTGGATATAGACGCTGACAGCGCCTTTTT
gi|259014734|gb|FJ959103.1|         AGATTAATAACCCATCATGGATCCTGTGTACGTGGACATAGACGCTGACAGCGCCTTTTT
gi|1032899593|gb|KX262988.1|        AGATTAATAACCCATCATGGATTCTGTGTACGTGGATATAGACGCTGACAGCGCCTTTTT
gi|954465478|gb|KR046227.1|         AGATTAATAACCCATCATGGATTCTGTGTACGTGGATATAGACGCTGACAGCGCCTTTTT
gi|1168909588|gb|KY703972.1|        AGATTAATAACCCATCATGGATTCTGTGTACGTGGATATAGACGCTGACAGCGCCTTTTT
gi|1333945209|gb|MF773569.1|        AGATTAATAACCCATCATGGATCTTGTGTACGTGGACATAGACGCTGACAGCGCCTTTTT
gi|1796551933|gb|MN402891.1|        AGATTAATAACCCATCATGGATCCTGTGTACGTGGATATAGACGCTGACAGCGCCTTTTT
gi|2329593809|gb|OL840906.1|        AGATTAATAACCCATCATGGATCCTGTGTACGTGGATATAGACGCTGACAGCGCCTTTTT
gi|1189411900|gb|MF001511.1|        AGATTAATAACCCATCATGGATTCTGTGTACGTGGATATAGACGCTGACAGCGCCTTTTT
gi|2489916086|gb|OP615961.1|        AGATTAATAACCCATCATGGATTCTGTGTACGTGGATATAGACGCTGACAGCGCCTTTTT
gi|1189411882|gb|MF001505.1|        AGATTAATAACCCATCATGGATTCTGTGTACGTGGATATAGACGCTGACAGCGCCTTTTT
gi|2620122017|gb|OR785139.1|        AGATTAATAACCCATCATGGATTCTGTGTACGTGGATATAGACGCTGACAGCGCCTTTTT
gi|1189411924|gb|MF001519.1|        AGATTAATAACCCATCATGGATTCTGTGTACGTGGATATAGACGCTGACAGCGCCTTTTT
gi|116047549|gb|DQ443544.2|         AGATTAATAACCCATCATGGATCCTGTGTACGTGGACATAGACGCTGACAGCGCCTTTTT
gi|2438228262|gb|ON262791.1|        AGATTAATAACCCATCATGGATCCTGTGTACGTGGACATAGACGCTGACAGCGCCTTTTT
gi|1621066058|gb|MK473628.1|        AGATTAATAACCCATCATGGATCCTGTGTACGTGGACATAGACGCTGACAGCGCCTTTTT
gi|124295579|gb|EF027135.1|         AGATTAATAACCCATCATGGATCCTGTGTACGTGGACATAGACGCTGACAGCGCCTTTTT
gi|171918919|gb|EU244823.2|         AGATTAATAACCCATCATGGATCCTGTGTACGTGGACATAGACGCTGACAGCGCCTTTTT
gi|221229058|gb|FJ000064.1|         AGATTAATAACCCATCATGGATCCTGTGTACGTGGACATAGACGCTGACAGCGCCTTTTT
gi|256403024|dbj|AB455493.1|        AGATTAATAACCCATCATGGATCCTGTGTACGTGGACATAGACGCTGACAGCGCCTTCTT
```

Accession number

```
                                    61                                                         120
gi|262410946|gb|FJ807896.1|         AGATTAATAACCCATCATGGATCCTGTGTACGTGGACATAGACGCTGACAGCGCCTTTTT
gi|1316030169|dbj|LC259094.1|       AGATTAAGAACCCATCATGGATCCTGTGTACGTGGACATAGACGCTGACAGCGCCTTTTT
gi|2563904643|gb|OR037308.1|        AGATTAATAACCCATCATGGATCCTGTGTACGTGGATATAGACGCTGACAGCGCCTTTTT
gi|2675378508|gb|PP319439.1|        AGATTAAGAACCCATCATGGATCCTGTGTACGTGGACATAGACGCTGACAGCGCCTTTTT
gi|1021315758|gb|KT308162.1|        AGATTAATAACCCATCATGGATTCTGTGTACGTGGATATAGACGCTGACAGCGCCTTTTT
gi|189409700|gb|EU703759.1|         AGATCAATAACCCATCATGGATTCTGTGTACGTGGATATAGACGCTGACAGCGCCTTTTT
gi|1333945188|gb|MF773562.1|        AGATTAATAACCCATCATGGATTCTGTGTACGTGGATATAGACGCTGACAGCGCCTTTTT
gi|541987890|gb|KF318729.1|         AGATTAATAACCCATCATGGATTCTGTGTACGTGGATATAGACGCTGACAGCGCCTTTTT
gi|615794507|gb|KJ451624.1|         AGATTAATAACCCATCATGGATTCTGTGTACGTGGATATAGACGCTGACAGCGCCTTTTT
gi|1843512200|gb|MN974212.1|        AGATTAATAACCCATCATGGATCCTGTGTACGTGGATATAGACGCTGACAGCGCCTTTTT
gi|1967465572|gb|MW042255.1|        AGATTAATAACCCATCATGGATCCTGTGTACGTGGACATAGACGCTGACAGCGCCTTTTT
gi|1843512188|gb|MN974208.1|        AGATTAATAACCCATCATGGATCCTGTGTACGTGGATATAGACGCTGACAGCGCCTTTTT
gi|1843512170|gb|MN974223.1|        AGATTAATAACCCATCATGGATCCTGTGTACGTGGATATAGACGCTGACAGCGCCTTTTT
gi|2438228268|gb|ON262793.1|        AGATTAATAACCCATCATGGATCCTGTGTACGTGGATATAGACGCTGACAGCGCCTTTTT
gi|2563904646|gb|OR037309.1|        AGATTAATAACCCATCATGGATCCTGTGTACGTGGATATAGACGCTGACAGCGCCTTTTT
gi|1621066055|gb|MK473627.1|        AGATTAATAACCCATCATGGATCCTGTGTACGTGGACATAGACGCTGACAGCGCCTTTTT
gi|156751972|gb|EU037962.1|         AGATTAATAACCCATCATGGATCCTGTGTACGTGGACATAGACGCTGACAGCGCCTTTTT
gi|801165883|gb|KP164869.1|         AGATTAATAACCCATCATGGATCCTGTGTACGTGGACATAGACGCTGACAGCGCCTTTTT
gi|1160525264|gb|KY751908.1|        AGATTAATAACCCATCATGGATCCTGTGTACGTGGACATAGACGCTGACAGCGCCTTTTT
gi|296124507|gb|HM045794.1|         AGATTAATAACCCATCATGGATCCTGTGTACGTGGACATAGACGCTGACAGCGCCTTTTT
gi|664818218|gb|KJ941050.1|         AGATTAATAACCCATCATGGATCCTGTGTACGTGGACATAGACGCTGACAGCGCCTTTTT
gi|429324003|emb|FN295485.3|        AGATTAATAACCCATCATGGATCCTGTGTACGTGGACATAGACGCTGACAGCGCCTTTTT
gi|2322557111|gb|OK562600.1|        AGATTAAGAACCCATCATGGATCCTGTGTACGTGGACATAGACGCTGACAGCGCCTTTTT
gi|296124529|gb|HM045802.1|         AGATTAATAACCCATCATGGATTCTGTGTACGTGGATATAGACGCTGACAGCGCCTTTTT
gi|124295582|gb|EF027136.1|         AGATTAATAACCCATCATGGATCCTGTGTACGTGGACATAGACGCTGACAGCGCCTTTTT
gi|2186567357|gb|OL999093.1|        AGATTAATAACCCATCATGGATCCTGTGTACGTGGATATAGACGCTGACAGCGCCTTTTT
gi|2438257538|gb|OQ148650.1|        AGATTAAGAACCCATCATGGATCCTGTGTACGTGGACATAGACGCTGACAGCGCCTTTTT
gi|2219863227|gb|ON009843.1|        AGATTAAGAACCCATCATGGATCCTGTGTACGTGGACATAGACGCTGACAGCGCCTTTTT
gi|1240390999|gb|MF774617.1|        AGATTAATAACCCATCATGGATCCTGTGTACGTGGACATAGACGCTGACAGCGCCTTTTT
gi|296124489|gb|HM045788.1|         AGATTAATAACCCATCATGGATTCTGTGTACGTGGACATAGACGCTGACAGCGCCTTTTT
gi|2186567214|gb|OL705486.1|        AGATTAATAACCCATCATGGATCCTGTGTACGTGGATATAGACGCTGACAGCGCCTTTTT
gi|296124564|gb|HM045814.1|         AGATTAATAACCCATCATGGATTCTGTGTACGTGGATATAGACGCTGACAGCGCCTTTTT
gi|1621066052|gb|MK473626.1|        AGATTAATAACCCATCATGGATTCTGTGTACGTGGACATAGACGCTGACAGCGCCTTTTT
gi|1992350182|gb|MT666072.1|        AGATTAAGAACCCATCATGGATCCTGTGTACGTGGACATAGACGCTGACAGCGCCTTTTT
gi|1240390993|gb|MF774615.1|        AGATTAATAACCCATCATGGATCCTGTGTACGTGGACATAGACGCTGACAGCGCCTTTTT
gi|1240391002|gb|MF774618.1|        AGATTAATAACCCATCATGGATCCTGTGTACGTGGACATAGACGCTGACAGCGCCTTTTT
gi|1147165987|gb|KY038947.2|        AGATTAAGAACCCATCATGGATCCTGTGTACGTGGACATAGACGCTGACAGCGCCTTTTT
gi|1677557251|gb|MH329303.1|        AGATTAATAACCCATCATGGATTCTGTGTACGTGGATATAGACGCTGACAGCGCCTTTTT
gi|1168909471|gb|KY703951.1|        AGATTAATAACCCATCATGGATTCTGTGTACGTGGATATAGACGCTGACAGCGCCTTTTT
gi|2216793899|gb|MW281311.1|        AGATTAAGAACCCATCATGGATCCTGTGTACGTGGACATAGACGCTGACAGCGCCTTTTT
gi|1728385259|gb|MK935343.1|        AGATTAAGAACCCATCATGGATCCTGTGTACGTGGACATAGACGCTGACAGCGCCTTTTT
gi|1677557239|gb|MH329299.1|        AGATTAATAACCCATCATGGATTCTGTGTACGTGGATATAGACGCTGACAGCGCCTTTTT
gi|1168909205|gb|KY703904.1|        AGATTAATAACCCATCATGGATTCTGTGTACGTGGATATAGACGCTGACAGCGCCTTTTT
gi|1032899626|gb|KX262993.1|        AGATTAATAACCCATCATGGATCCTGTGTACGTGGACATAGACGCTGACAGCGCCTTTTT
gi|2484235380|gb|OQ605439.1|        AGATTAACAACCCATCATGGATCCTGTGTACGTGGACATAGACGCTGACAGCGCCTTTTT
gi|1032899599|gb|KX262989.1|        AGATTAATAACCCATCATGGATCCTGTGTACGTGGACATAGACGCTGACAGCGCCTTTTT
gi|1798045089|gb|MK370032.1|        AGATTAATAACCCATCATGGATCCTGTGTACGTGGACATAGACGCTGACAGCGCCTTTTT
gi|2186567343|gb|OL999091.1|        AGATTAATAACCCATCATGGATCCTGTGTACGTGGACATAGACGCTGACAGCGCCTTTTT
gi|1621066043|gb|MK473623.1|        AGATTAATAACCCATCATGGATCCTGTGTACGTGGACATAGACGCTGACAGCGCCTTTTT
gi|2489916092|gb|OP615963.1|        AGATTAAGAACCCATCATGGATCCTGTGTACGTGGACATAGACGCTGACAGCGCCTTTTT
gi|2186567294|gb|OL979154.1|        AGATTAATAACCCATCATGGATCCTGTGTACGTGGATATAGACGCTGACAGCGCCTTTTT
gi|1992350185|gb|MT666073.1|        AGATTAAGAACCCATCATGGATCCTGTGTACGTGGACATAGACGCTGACAGCGCCTTTTT
gi|1626774529|gb|MH124576.1|        AGATTAAGAACCCATCATGGATCCTGTGTACGTGGACATAGACGCTGACAGCGCCTTTTT
gi|124295597|gb|EF027141.1|         AGATTAATAACCCATCATGGATTCTGTGTACGTGGACATAGACGCTGACAGCGCCTTTTT
gi|745698484|gb|KP003812.1|         AGATTAAGAACCCATCATGGATCCTGTGTACGTGGACATAGACGCTGACAGCGCCTTTTT
gi|1778598293|gb|MK040569.1|        AGATTAATAACCCATCATGGATCCTGTGTACGTGGATATAGACGCTGACAGCGCCTTTTT
gi|1240391005|gb|MF774619.1|        AGATTAATAACCCATCATGGATCCTGTGTACGTGGACATAGACGCTGACAGCGCCTTTTT
gi|1798045083|gb|MK370030.1|        AGATTAATAACCCATCATGGATCCTGTGTACGTGGACATAGACGCTGACAGCGCCTTTTT
gi|1316030139|dbj|LC259084.1|       AGATTAATAACCCATCATGGATTCTGTGTACGTGGATATAGACGCTGACAGCGCCTTTTT
gi|2118777267|gb|MZ443814.1|        AGATTAATAACCCATCATGGATCCTGTGTACGTGGATATAGACGCTGACAGCGCCTTTTT
gi|1861718970|gb|MT526804.1|        AGATTAATAACCCATCATGGATCCTGTGTACGTGGACATAGACGCTGACAGCGCCTTTTT
gi|1032899586|gb|KX262987.1|        AGATTAATAACCCATCATGGATTCTGTGTACGTGGATATAGACGCTGACAGCGCCTTTTT
gi|1240390988|gb|MF774613.1|        AGATTAATAACCCATCATGGATCCTGTGTACGTGGACATAGACGCTGACAGCGCCTTTTT
gi|1168909546|gb|KY703965.1|        AGATTAATAACCCATCATGGATTCTGTGTACGTGGATATAGACGCTGACAGCGCCTTTTT
gi|428670855|emb|HE806461.1|        AGATTAATAACCCATCATGGATTCTGTGTACGTGGATATAGACGCTGACAGCGCCTTTTT
gi|1685826244|gb|MH359141.1|        AGATTAATAACCCATCATGGATTCTGTGTACGTGGATATAGACGCTGACAGCGCCTTTTT
gi|2018315610|gb|MW574902.1|        AGATTAATAACCCATCATGGATCCTGTGTACGTGGACATAGACGCTGACAGCGCCTTTTT
gi|659902278|gb|KJ679578.1|         AGATTAATAACCCATCATGGATCCTGTGTACGTGGACATAGACGCTGACAGCGCCTTTTT
gi|2438257514|gb|OQ148642.1|        AGATTAAGAACCCATCATGGATCCTGTGTACGTGGACATAGACGCTGACAGCGCCTTTTT
gi|1279118184|gb|MG280943.1|        AGATTAAGAACCCATCATGGATCCTGTGTACGTGGACATAGACGCTGACAGCGCCTTTTT
gi|124295576|gb|EF027134.1|         AGATTAATAACCCATCATGGATCCTGTGTACGTGGACATAGACGCTGACAGCGCCTTTTT
gi|296124486|gb|HM045787.1|         AGATTAATAACCCATCATGGATTCTGTGTACGTGGATATAGACGCTGACAGCGCCTTTTT
gi|296124549|gb|HM045809.1|         AGATTAAGAACCCATCATGGATCCTGTGTACGTGGACATAGACGCTGACAGCGCCTTTTT
gi|296124501|gb|HM045792.1|         AGATTAAGAACCCATCATGGATCCTGTGTACGTGGACATAGACGCTGACAGCGCCTTTTT
gi|124295588|gb|EF027138.1|         AGATTAATAACCCATCATGGATCCTGTGTACGTGGACATAGACGCTGACAGCGCCTTTTT
gi|2186567333|gb|OL979157.1|        AGATTAATAACCCATCATGGATCCTGTGTACGTGGATATAGACGCTGACAGCGCCTTTTT
gi|1859767498|gb|MK993755.1|        AGATTAAGAACCCATCATGGATCCTGTGTACGTGGACATAGACGCTGACAGCGCCTTTTT
```

Accession number 61 – 120

gi|1168907703|gb|KY680385.1| AGATTAATAACCCATCATGGATTCTGTGTACGTGGATATAGACGCTGACAGCGCCTTTTT
gi|1861718958|gb|MT526800.1| AGATTAATAACCCATCATGGATCCTGTGTACGTGGACATAGACGCTGACAGCGCCTTTTT
gi|659902275|gb|KJ679577.1| AGATTAAGAACCCATCATGGATCCTGTGTACGTGGACATAGACGCTGACAGCGCCTTTTT
gi|296124523|gb|HM045800.1| AGATTAATAACCCATCATGGATTCTGTGTACGTGGATATAGACGCTGACAGCGCCTTTTT
gi|262073965|gb|FJ513654.1| AGATTAATAACCCATCATGGATCCTGTGTACGTGGACATAGACGCTGACAGCGCCTTTTT
gi|262073947|gb|FJ513628.1| AGATTAATAACCCATCATGGATCCTGTGTACGTGGACATAGACGCTGACAGCGCCTTTTT
gi|296124495|gb|HM045790.1| AGATTAATAACCCATCATGGATTCTGTGTACGTGGATATAGACGCTGACAGCGCCTTTTT
gi|262073968|gb|FJ513657.1| AGATTAATAACCCATCATGGATCCTGTGTACGTGGACATAGACGCTGACAGCGCCTTTTT
gi|296124541|gb|HM045806.1| AGATTAAGAACCCATCATGGATCCTGTGTACGTGGACATAGACGCTGACAGCGCCTTTTT
gi|2484235365|gb|OQ605434.1| AGATTAACAACCCATCATGGATCCTGTGTACGTGGACATAGACGCTGACAGCGCCTTTTT
gi|1859767502|gb|MK993757.1| AGATTAAGAACCCATCATGGATCCTGTGTACGTGGACATAGACGCTGACAGCGCCTTTTT
gi|2577286133|gb|OR283254.1| AGATTAAGAACCCATCATGGATCCTGTGTACGTGGACATAGACGCTGACAGCGCCTTTTT
gi|371985830|gb|JN558834.1| AGATTAATAACCCATCATGGATCCTGTGTACGTGGACATAGACGCTGACAGCGCCTTTTT
gi|2484235386|gb|OQ605441.1| AGATTAACAACCCATCATGGATCCTGTGTACGTGGACATAGACGCTGACAGCGCCTTTTT
gi|288572683|gb|FJ445426.2| AGATTAATAACCCATCATGGATCCTGTGTACGTGGACATAGACGCTGACAGCGCCTTTTT
gi|1992350179|gb|MT666071.1| AGATTAAGAACCCATCATGGATCCTGTGTACGTGGACATAGACGCTGACAGCGCCTTTTT
gi|288572704|gb|FJ445443.2| AGATTAATAACCCATCATGGATCCTGTGTACGTGGACATAGACGCTGACAGCGCCTTTTT
gi|2674493701|gb|PP193832.1| AGATTAATAACCCATCATGGATCCTGTGTACGTGGATATAGACGCTGACAGCGCCTTTTT
gi|2322557114|gb|OK562601.1| AGATTAAGAACCCATCATGGATCCTGTGTACGTGGACATAGACGCTGACAGCGCCTTTTT
gi|1565065272|gb|MK120202.1| AGATTAATAACCCATCATGGATCCTGTGTACGTGGACATAGACGCTGACAGCGCCTTTTT
gi|1565065269|gb|MK120201.1| AGATTAATAACCCATCATGGATCCTGTGTACGTGGACATAGACGCTGACAGCGCCTTTTT
gi|1559671006|gb|MH423806.1| AGATTAATAACCCATCATGGATCCTGTGTACGTGGACATAGACGCTGACAGCGCCTTTTT
gi|2438257517|gb|OQ148643.1| AGATTAAGAACCCATCATGGATCCTGTGTACGTGGACATAGACGCTGACAGCGCCTTTTT
gi|655168290|gb|KJ796852.1| AGATTAATAACCCATCATGGATCCTGTGTACGTGGACATAGACGCTGACAGCGCCTTTTT
gi|2118777264|gb|MZ443813.1| AGATTAATAACCCATCATGGATCCTGTGTACGTGGATATAGACGCTGACAGCGCCTTTTT
gi|296124510|gb|HM045795.1| AGATTAAGAACCCATCATGGATCCTGTGTACGTGGACATAGACGCTGACAGCGCCTTTTT
gi|124295594|gb|EF027140.1| AGATTAATAACCCATCATGGATTCTGTGTACGTGGACATAGACGCTGACAGCGCCTTTTT
gi|1168909139|gb|KY703893.1| AGATTAATAACCCATCATGGATTCTGTGTACGTGGATATAGACGCTGACAGCGCCTTTTT
gi|1171733075|gb|KX881784.1| AGATTAATAACCCATCATGGATCCTGTGTACGTGGACATAGACGCTGACAGCGCCTTTTT
gi|1621066040|gb|MK473622.1| AGATTAATAACCCATCATGGATCCTGTGTACGTGGACATAGACGCTGACAGCGCCTTTTT
gi|2484235389|gb|OQ605442.1| AGATTAACAACCCATCATGGATCCTGTGTACGTGGACATAGACGCTGACAGCGCCTTTTT
gi|1032899645|gb|KX262996.1| AGATTAAGAACCCATCATGGATCCTGTGTACGTGGACATAGACGCTGACAGCGCCTTTTT
gi|1565065260|gb|MK120198.1| AGATTAATAACCCATCATGGATCCTGTGTACGTGGACATAGACGCTGATAGCGCCTTTTT
gi|1032899632|gb|KX262994.1| AGATTAATAACCCATCATGGATTCTGTGTACGTGGATATAGACGCTGACAGCGCCTTTTT
gi|1621066049|gb|MK473625.1| AGATTAATAACCCATCATGGATCCTGTGTACGTGGACATAGACGCTGACAGCGCCTTTTT
gi|1154340972|gb|KY575574.1| AGATTAAGAACCCATCATGGATCCTGTGTACGTGGACATAGACGCTGACAGCGCCTTTTT
gi|2438257478|gb|OQ148630.1| AGATTAAGAACCCATCATGGATCCTGTGTACGTGGACATAGACGCTGACAGCGCCTTTTT
gi|1168909576|gb|KY703970.1| AGATTAATAACCCATCATGGATTCTGTGTACGTGGATATAGACGCTGACAGCGCCTTTTT
gi|1168909314|gb|KY703923.1| AGATTAATAACCCATCATGGATTCTGTGTACGTGGATATAGACGCTGACAGCGCCTTTTT
gi|2438731903|gb|MW588418.1| AGATTAATAACCCATCATGGATCCTGTGTACGTGGACATAGACGCTGACAGCGCCTTTTT
gi|1677557221|gb|MH329293.1| AGATTAATAACCCATCATGGATTCTGTGTACGTGGATATAGACGCTGACAGCGCCTTTTT
gi|1032899652|gb|KX262997.1| AGATTAATAACCCATCATGGATCCTGTGTACGTGGACATAGACGCTGACAGCGCCTTTTT
gi|1168909527|gb|KY703961.1| AGATTAATAACCCATCATGGATTCTGTGTACGTGGATATAGACGCTGACAGCGCCTTTTT
gi|655168284|gb|KJ796849.1| AGATTAATAACCCATCATGGATCCTGTGTACGTGGACATAGACGCTGACAGCGCCTTTTT
gi|655168282|gb|KJ796848.1| AGATTAATAACCCATCATGGATCCTGTGTACGTGGACATAGACGCTGACAGCGCCTTTTT
gi|1733482778|gb|MK551553.1| AGATTAATAACCCATCATGGATCCCGTGTACGTGGACATAGACGCTGACAGCGCCTTTTT
gi|2438257442|gb|OQ148618.1| AGATTAATAACCCATCATGGATTCTGTGTACGTGGATATAGACGCTGACAGCGCCTTTTT
gi|1199750720|gb|KX619425.1| AGATTAATAACCCATCATGGATCCTGTGTACGTGGACATAGACGCTGACAGCGCCTTTTT
gi|1621066076|gb|MK473634.1| AGATTAATAACCCATCATGGATCCTGTGTACGTGGACATAGACGCTGACAGCGCCTTTTT
gi|1733482772|gb|MK518340.1| AGATTAAGAACCCATCATGGATCCTGTGTACGTGGACATAGACGCTGACAGCGCCTTTTT
gi|2484235422|gb|OQ605453.1| AGATTAACAACCCATCATGGATCCTGTGCACGTGGACATAGACGCTGACAGCGCCTTTTT
gi|1626774541|gb|MH124580.1| AGATTAATAACCCATCATGGATCCTGTGTACGTGGACATAGACGCTGACAGCGCCTTTTT
gi|2715015475|gb|PP599025.1| AGATTAATAACCCATCATGGATCCTGTGTACGTGGATATAGACGCTGACAGCGCCTTTTT
gi|1983384050|gb|MT495605.1| AGATTAATAACCCATCATGGATCCTGTGTACGTGGATATAGACGCTGACAGCGCCTTTTT
gi|2438731973|gb|MW588419.1| AGATTAATAACCCATCATGGATCCTGTGTACGTGGACATAGACGCTGACAGCGCCTTTTT
gi|2484235425|gb|OQ605454.1| AGATTAACAACCCATCATGGATCCTGTGCACGTGGACATAGACGCTGACAGCGCCTTTTT
gi|380467950|gb|JQ067624.1| AGATTAAGAACCCATCATGGATCCTGTGTACGTGGACATAGACGCTGACAGCGCCTTTTT
gi|1954890103|gb|MT526903.1| AGATTAAGAACCCATCATGGATCCTGTGTACGTGGACATAGACGCTGACAGCGCCTTTTT
gi|1199750723|gb|KX619426.1| AGATTAATAACCCATCATGGATCCTGTGTACGTGGACATAGACGCTGACAGCGCCTTTTT
gi|1626774550|gb|MH124583.1| AGATTAATAACCCATCATGGATCCTGTGTACGTGGACATAGACGCTGACAGCGCCTTTTT
gi|1626774547|gb|MH124582.1| AGATTAATAACCCATCATGGATCCTGTGTACGTGGACATAGACGCTGACAGCGCCTTTTT
gi|2316015179|gb|MZ703086.1| AGATTAAGAACCCATCATGGATCCTGTGTACGTGGACATAGACGCTGACAGCGCCTTTTT
gi|1733482775|gb|MK551552.1| AGATTAATAACCCATCATGGATCCTGTGTACGTGGACATAGACGCTGACAGCGCCTTTTT
gi|1621066046|gb|MK473624.1| AGATTAATAACCCATCATGGATCCTGTGTACGTGGACATAGACGCTGACAGCGCCTTTTT
gi|2186567348|gb|OL999092.1| AGATTAATAACCCATCATGGATCCTGTGTACGTGGATATAGACGCTGACAGCGCCTTTTT
gi|1168909278|gb|KY703917.1| AGATCAATAACCCATCATGGATTCTGTGTACGTGGATATAGACGCTGACAGCGCCTTTTT
gi|296124591|gb|HM045823.1| AGATTAAGAACCCATCATGGATCCTGTGTACGTGGACATAGACGCTGACAGCGCCTTTTT
gi|296124477|gb|HM045784.1| AGATTAAGAACCCATCATGGATCCTGTGTACGTGGACATAGACGCTGACAGCGCCTTTTT
gi|1778598299|gb|MK040571.1| AGATTAATAACCCATCATGGATCCTGTGTACGTGGACATAGACGCTGACAGCGCCTTTTT
gi|655168288|gb|KJ796851.1| AGATTAATAACCCATCATGGATCCTGTGTACGTGGACATAGACGCTGACAGCGCCTTTTT
gi|280983623|gb|GU199350.1| AGATTAATAACCCATCATGGATCCTGTGTACGTGGACATAGACGCTGACAGCGCCTTTTT
gi|1168909157|gb|KY703896.1| AGATTAATAACCCATCATGGATTCTGTGTACGTGGATATAGACGCTGACAGCGCCTTTTT
gi|2287617414|gb|MW557660.1| AGATTAATAACCCATCATGGATCCTGTGTACGTGGATATAGACGCTGACAGCGCCTTTTT
gi|2154025537|gb|MW473668.1| AGATTAATAACCCATCATGGATTCTGTGTACGTGGACATAGACGCTGACAGCGCCTTTTT
gi|2484235401|gb|OQ605446.1| AGATTAACAACCCATCATGGATCCTGTGTACGTGGACATAGACGCTGACAGCGCCTTTTT
gi|1168907836|gb|KY680408.1| AGATTAACAACCCATCATGGATTCTGTGTACGTGGATATAGACGCTGACAGCGCCTTTTT

Supplementary data 2: CHIKV predicted RNA structure (nts 1-300) in **A)** wild type **B) m**utant ^{63}CAACUU^{68} and **C)** mutant $^{A}67^{G}$. RNA structure mapped free energy minimization using Pfold on default settings.

A) Wild type

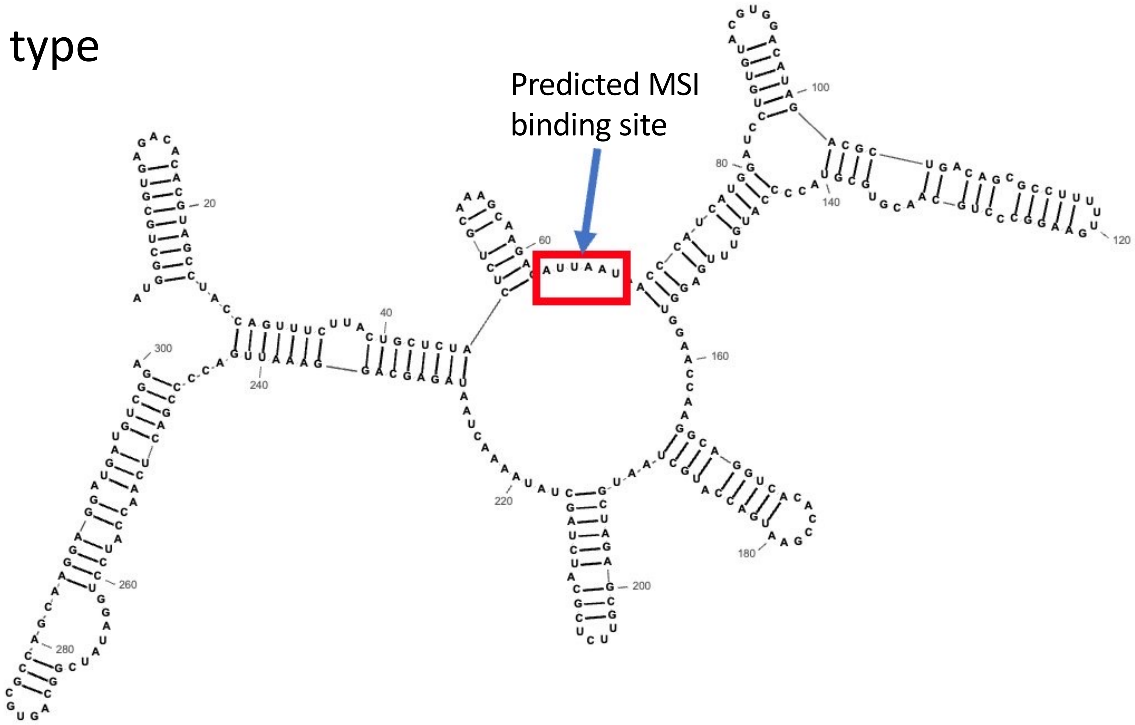

B) Mutant ^{63}CAACUU^{68}

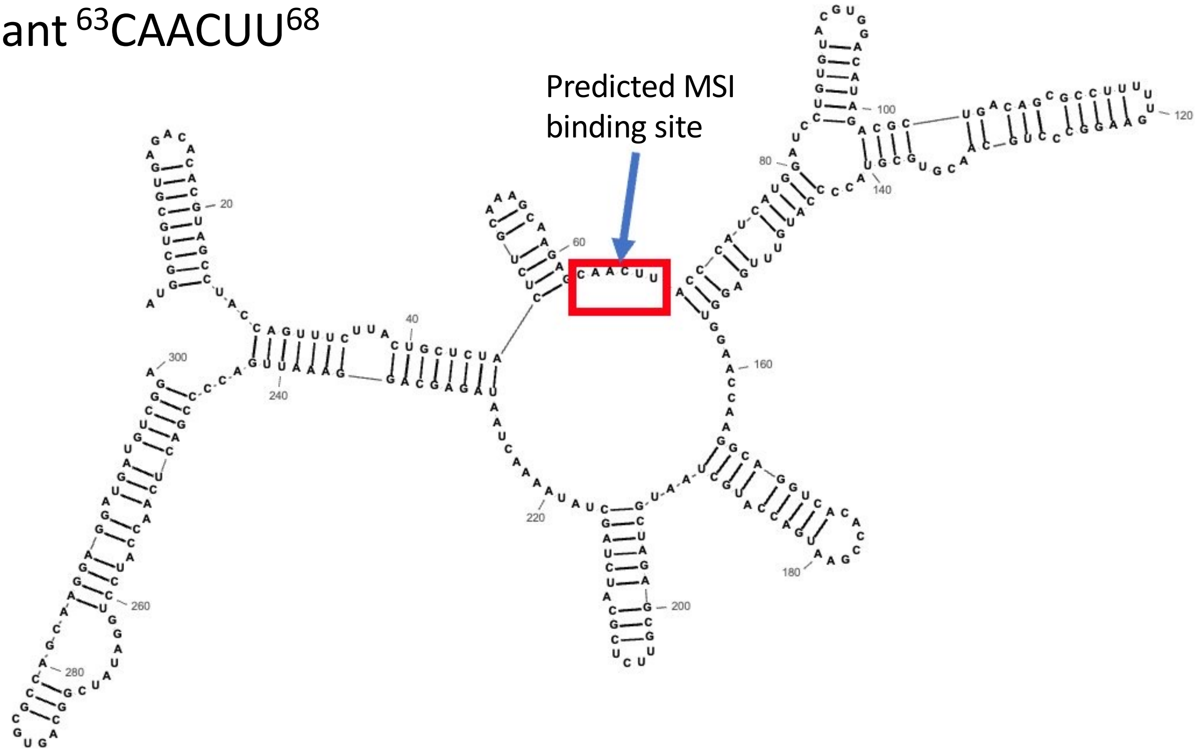

Supplementary data 2: CHIKV predicted RNA structure (nts 1-300) in **A)** wild type **B)** **m**utant ^{63}CAACUU^{68} and **C)** mutant ^{A}67^{G}. RNA structure mapped free energy minimization using Pfold on default settings.

C) Mutant ^{A}67^{G}

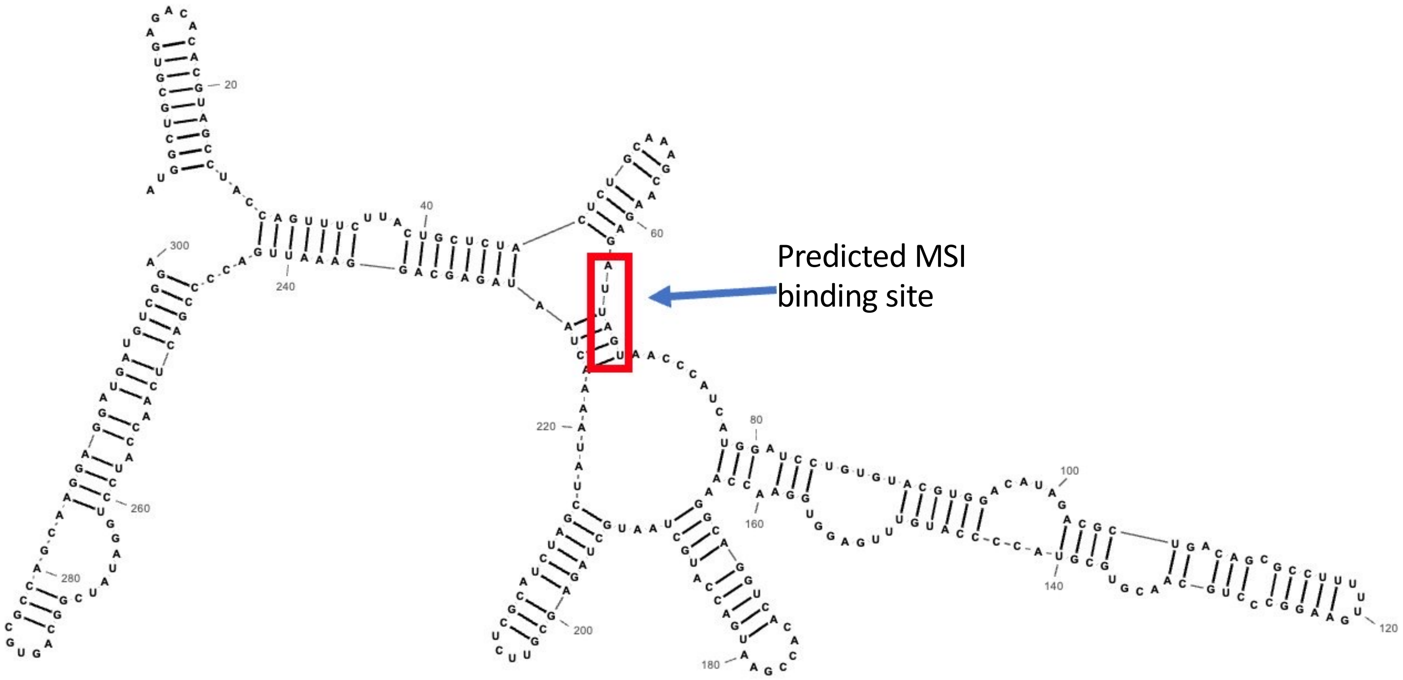

Supplementary data 3: Primers for the reverse transcription and quantitative PCRs for CHIKV strand-specific detection.

**CHIKV (-) strand detection**	**PCR**	**Primer sequence (5'-3')**
**CHIKV FT tag T**	reverse transcription	GGC AGT ATC GTG AAT TCG ATG CGA CAC GGA GAC GCC AAC ATT
**Tag T**	quantitative	GGC AGT ATC GTG AAT TCG ATG C
**CHIKV R T**	quantitative	AAT AAA TCA TAA GTC TGC TCT CTG TCT ACA TGA
**CHIKV (+) strand detection**	PCR	Primer sequence (5'-3')
**CHIKV RT tag T**	reverse transcription	GGC AGT ATC GTG AAT TCG ATG CGT CTG CTC TCT GTC TAC ATG A
**CHIKV F T**	quantitative	AAT AAA TCA TAA GAC ACG GAG ACG CCA ACA TT
**Tag T**	quantitative	see above

Supplementary data 4: Expression and purification of MSI-2 Coomassie stained PAGE analysis following **A)** His Tag and **B)** Ion exchange chromatography. **C)** Ion exchange purified MSI-2 (8-193) analyzed by western blot, relative to total protein extracted from RD and Huh7 cells.

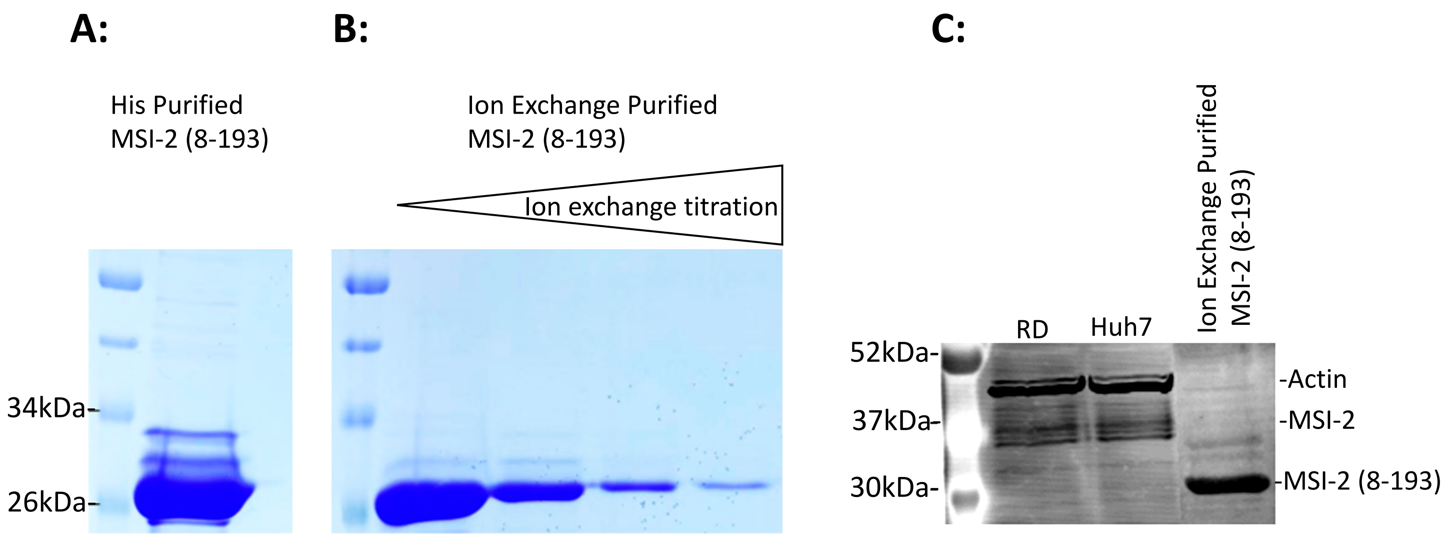

Supplementary data 5: MTT cytotoxicity assay for Ro 08-2750 in RD cells across a titration of 0, 05, 1, 3, 5 10 and 20uM. N=3, error bars represent standard error from the mean.

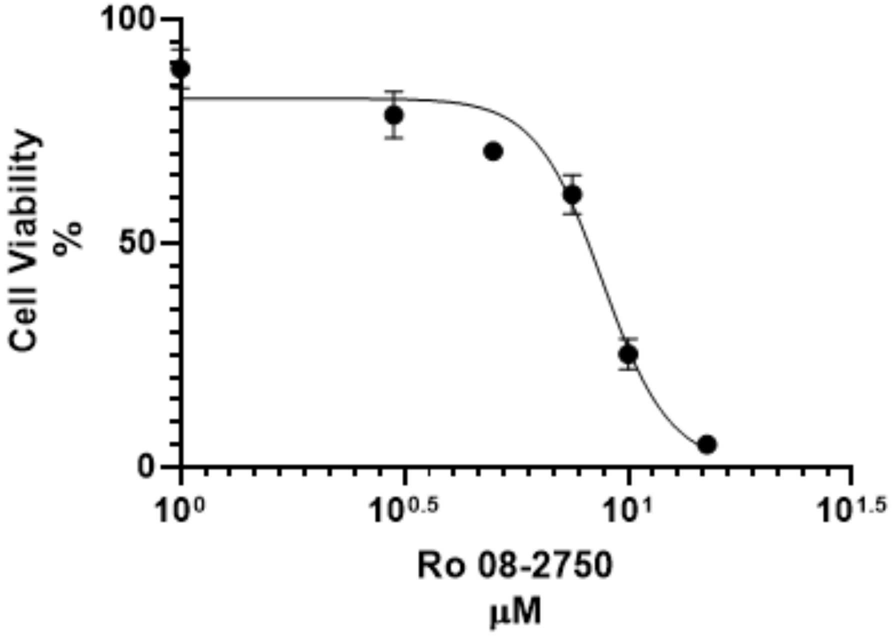

Supplementary data 6: A) MSI-1 and **B**) MSI-2 expression in RD and Huh7 cell lysate analyzed by western blot.

A)

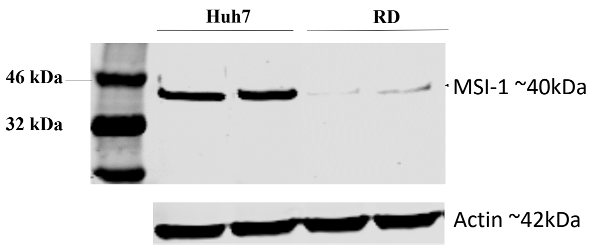

B)

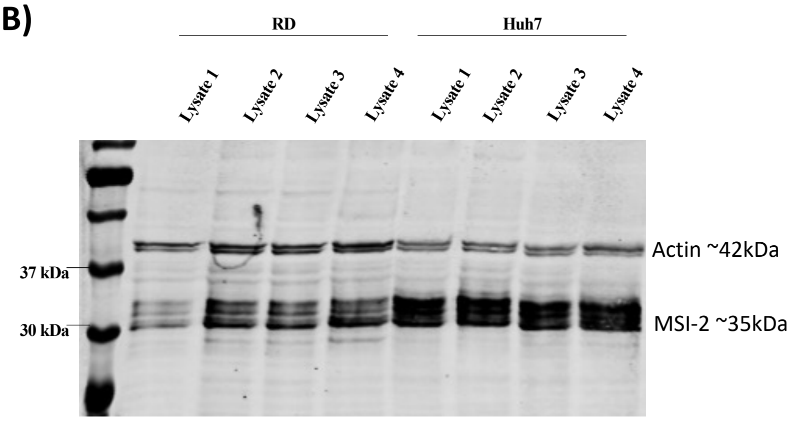

Supplementary data 7: Co-inhibition of MSI-2 and MSI-1 by siRNA in **A)** and **B)** Huh7 cells and **C)** RD cells

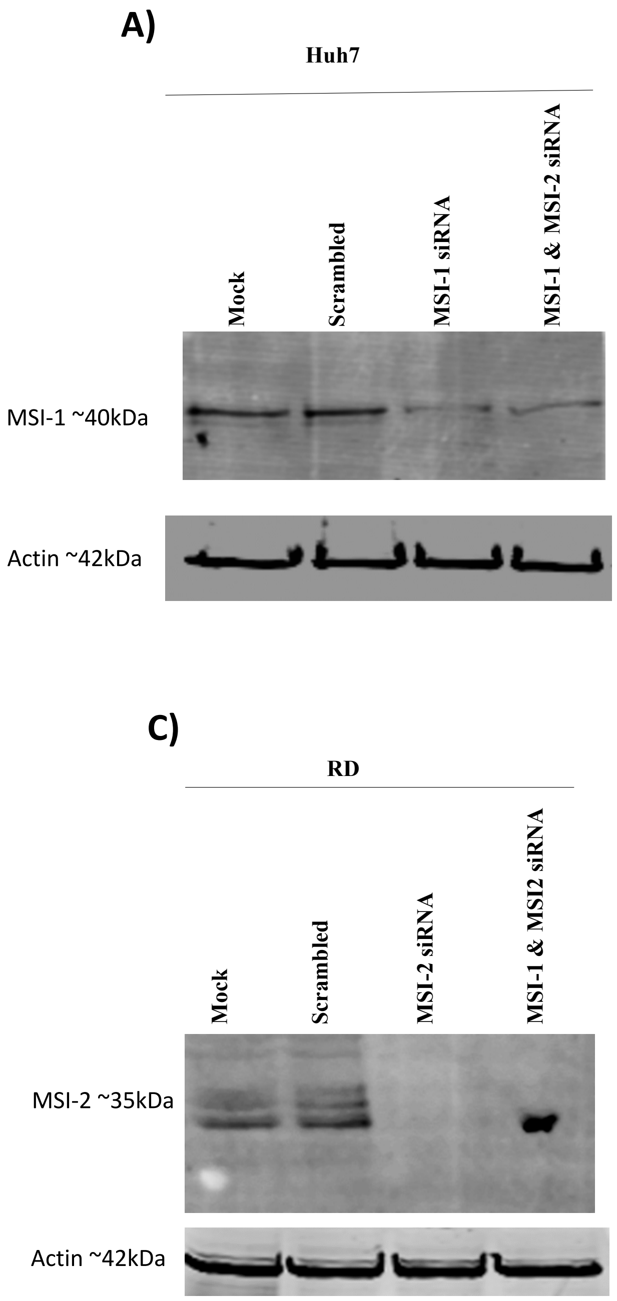

Supplement: gkae619_Supplemental_File [file gkae619_supplemental_file.pdf]
